# Alcohol use disorders and the risk of progression of liver disease in people with hepatitis C virus infection – a systematic review

**DOI:** 10.1186/s13011-020-00287-1

**Published:** 2020-06-30

**Authors:** Laura Llamosas-Falcón, Kevin D. Shield, Maya Gelovany, Jakob Manthey, Jürgen Rehm

**Affiliations:** 1 Preventive Medicine and Public Health, Preventive Medicine, Universitary Hospital “12 de Octubre”, Avda de Córdoba s/n 28041, Madrid, Spain; 2 Institute for Mental Health Policy Research, Centre for Addiction and Mental Health, 33 Russell Street, Room T420, Toronto, Ontario M5S 2S1 Canada; 3 Dalla Lana School of Public Health, University of Toronto, 155 College Street, Toronto, Ontario M5T 1P8 Canada; 4 Center for Interdisciplinary Addiction Research (ZIS), Department of Psychiatry and Psychotherapy, University Medical Center Hamburg-Eppendorf (UKE), Martinistraße 52, 20246 Hamburg, Germany; 5 Institute of Clinical Psychology and Psychotherapy & Center of Clinical Epidemiology and Longitudinal Studies (CELOS), Technische Universität Dresden, Chemnitzer Str. 46, 01187 Dresden, Germany; 6 Campbell Family Mental Health Research Institute, Centre for Addiction and Mental Health, 33 Russell Street, Toronto, Ontario M5T 2S1 Canada; 7 Faculty of Medicine, Institute of Medical Science, University of Toronto, Medical Sciences Building, 1 King’s College Circle, Room 2374, Toronto, Ontario M5S 1A8 Canada; 8 Department of Psychiatry, University of Toronto, 250 College Street, 8th floor, Toronto, Ontario M5T 1R8 Canada; 9 Department of International Health Projects, Institute for Leadership and Health Management, I.M. Sechenov First Moscow State Medical University, Trubetskaya str., 8, b. 2, Moscow, Russian Federation 119992

**Keywords:** Alcohol, Alcohol-use-disorders, Hepatitis C virus infection, Liver-disease progression, Liver cirrhosis, Decompensated liver cirrhosis, Meta-analysis

## Abstract

Liver cirrhosis and other chronic liver diseases are usually compartmentalized into separate categories based on etiology (e.g., due to alcohol, virus infection, etc.), but it is important to study the intersection of, and possible interactions between, risk factors. The aim of this study is to summarize evidence on the association between alcohol use disorders (AUDs) and decompensated liver cirrhosis and other complications in patients with chronic Hepatitis C virus (HCV) infection. A systematic search of epidemiological studies was conducted using Ovid Medline databases in accordance with the Preferred Reporting Items for Systematic Reviews and Meta-Analyses criteria. Relative Risk estimates were combined using random-effects meta-analyses. The proportion of cases with liver disease progression that could be avoided if no person with a chronic HCV infection had an AUD was estimated using an attributable fraction methodology. A total of 11 studies fulfilled the inclusion criteria, providing data from 286,641 people with chronic HCV infections, of whom 63,931 (22.3%) qualified as having an AUD. Using decompensated liver cirrhosis as the outcome for the main meta-analysis (*n* = 7 unique studies), an AUD diagnosis was associated with a 3.3-fold risk for progression of liver disease among people with a chronic HCV infection (95% Confidence Interval (CI): 1.8–4.8). In terms of population-attributable fractions, slightly less than 4 out of 10 decompensated liver cirrhosis cases were attributable to an AUD: 35.2% (95% CI: 16.2–47.1%). For a secondary analyses, all outcomes related to liver disease progression were pooled (i.e., liver deaths or cirrhosis in addition to decompensated liver cirrhosis), which yielded a similar overall effect (*n* = 13 estimates; OR = 3.7; 95% CI: 2.2–5.3) and a similar attributable fraction (39.3%; 95% CI: 21.9–50.4%). In conclusion, AUDs were frequent in people with chronic HCV infections and contributed to worsening the course of liver disease. Alcohol use and AUDs should be assessed in patients who have liver disease of any etiology, and interventions should be implemented to achieve abstinence or to reduce consumption to the greatest possible extent.

## Main text

### The compartmentalization of liver cirrhosis

Both epidemiologically and clinically, liver cirrhosis and other chronic liver diseases are generally compartmentalized into separate categories based on their etiology. Thus, the Global Health Estimates [1] or the Global Burden of Disease Study [2] give prevalence, incidence, and mortality rates for them in different categories, separating cirrhosis and other chronic liver diseases by etiology: alcohol use, hepatitis B virus infection, hepatitis C virus (HCV) infection, non-alcoholic steatohepatitis, and other causes. Similar differentiation can be found clinically and in the International Classification of Diseases [3], even though there have been some calls to change this system [4, 5]. This contribution will not focus on the logic of current classifications, but will look at the intersection between two of these seemingly separate categories, i.e. liver diseases due to alcohol use and due to HCV infection.

### Aims of the current contribution

Based on a systematic literature search, we examined the role of heavy alcohol use—as operationalized via alcohol use disorders (AUDs) [6, 7]—on the progression of liver disease in people with chronic HCV infection. We hypothesized, based on a recent large-scale retrospective cohort study on all hospitalizations in France [8], that a large proportion of complications arising over the course of liver disease in people with HCV infection is attributable to AUD. The main outcome was “decompensated liver cirrhosis”, defined as an acute deterioration in liver function in a patient with cirrhosis, and characterized by jaundice, ascites, hepatic encephalopathy, hepatorenal syndrome and/or variceal hemorrhage [9, 10]. We will summarize the link between AUDs and decompensated liver cirrhosis and other complications of liver disease in people with HCV infection, by pooling relevant studies using meta-analytical techniques [11].

## Methods

### Systematic search and inclusion/exclusion

**Fig. 1:**
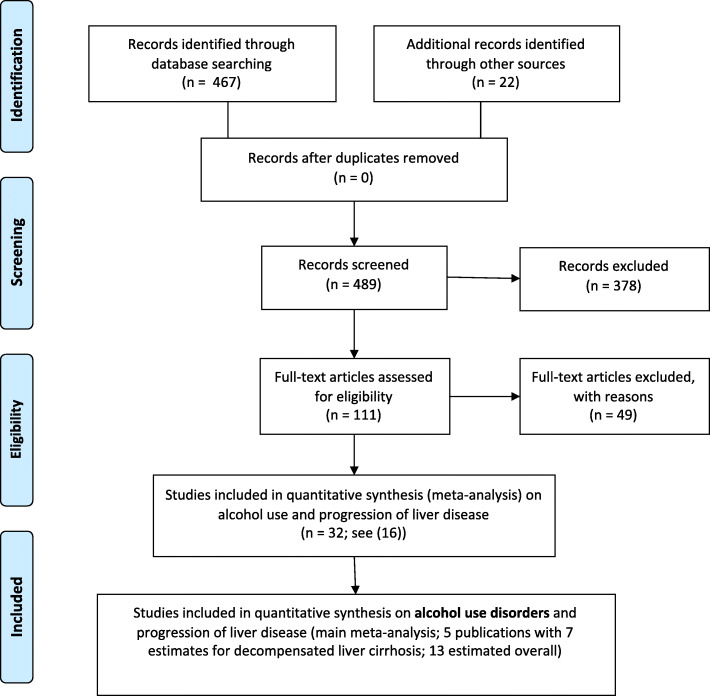
Systematic search results and selection process of studies for the meta-analyses

As a first step, we conducted a systematic search of epidemiological studies on the relationship between alcohol use and progression of liver disease due to HCV, using Ovid Medline databases, and applying the Preferred Reporting Items for Systematic Reviews and Meta-Analyses (PRISMA) criteria [12]. The exact search terms can be found in the Supplementary Materials (Table S1), but we looked for cohort or case-control studies (for definition, see [13]) of people with chronic HCV infections; with at least two different forms of alcohol use (e.g., alcohol use yes/no; AUD yes/no); and a verified indicator of progression of liver disease (e.g., progression of fibrosis, progression of cirrhosis to decompensated cirrhosis; liver death). This search was initially conducted on July 28, 2019 and updated on December 22, 2019, with the updated search yielding 467 references (see Fig. 1).

In addition, we conducted a search on systematic reviews and meta-analyses on this theme and searched for relevant articles this way (in particular [14–16], see the latter for further detail on the search strategy). As the second step, all articles which included AUDs as exposure were selected as the basis of the analyses of this paper.

For measurement of AUDs, we included the following: hospital record, other record in the healthcare or government database (e.g., registration), physician judgement, standardized measurement with a validated instrument such as the World Health Organization (WHO) Composite International Diagnostic Interview [17], self-report about major criteria of AUDs (for a general discussion of criteria, see [6, 18]). While chronic heavy drinking above 80 g pure alcohol per day before or at some point over the course of the disease could be part of the inclusion criteria (e.g., [19]), it alone did not suffice for inclusion in the study, as no generally accepted quantitative threshold for AUDs based on level of drinking has yet been established [7, 20]. We also excluded studies solely based on screening instruments, such as the CAGE [21] or the AUDIT [22].

All articles were screened by two of the authors (either LLF, MG, or JR). An overview of the selection process can be found in Fig. 1 [23]. All included studies were extracted by two authors (LLF and JR) for key information (study characteristics: title, authors, year published, country, study design, year of study; study population: total number of patients with HCV infection; participant details: mean age, sex, HIV coinfection, number of people with AUD; measurement of AUD; risk relations: relative risk indicator, confidence intervals, *p* value, adjustments (other covariates); and outcomes).

### Statistical methods

Relative Risk estimates (either Odds Ratios, Relative Risks or Hazard Ratios [13, 24]) were taken directly from the respective article or calculated based on a 2*2 table [25] or, in one case, using the methodology specified by Hamling et al. [26].

In the main analysis, only decompensated liver cirrhosis or its main constituents (defined above [9, 10]) as confirmed from medical records, hospitalization or death was chosen as the endpoint (*n* = 7 estimates from 5 studies). A random effect meta-analysis [27], accounting for the hierarchical structure of the data (three estimates from one study) was conducted [28].

In a secondary analysis, we repeated the random-effect meta-analyses for all estimates of progression of liver disease, i.e. applied a looser inclusion criterion. Here, the following endpoints were including as well (see also Table 1: advanced fibrosis, liver cirrhosis, liver deaths). For both models, we tested if adjustment for important covariates has an impact on the overall effect. Cochran’s Q and the I-squared statistic were used as indicators for heterogeneity [38, 39].

**Table 1:** Characteristics of studies included in the meta-analyses

Reference	Country	Type of study	Years of Study	N (N of outcome)	Measurement of AUD (N of people with AUD)	Outcome	Risk Relations^a^ Adjusted yes/no
Alavi et al., 2018 [29]	Australia	Cohort study based on record linkage	1995–2013 (1995–2012 HCV notifications)	82,526 (2559)	Non-liver-related hospitalization due to alcohol use disorders 2001–2013 (prior to outcome) (*N* = 14,797)	First-time hospitalization (or death, if no prior hospitalization) due to decompensated cirrhosis	HR: 3.68 (3.38–4.00) y
Alavi et al., 2018 [29]	Canada	Cohort study based on record linkage	1995–2012 (1995–2011 HCV notifications)	55,873 (2443)	Non-liver-related hospitalization due to alcohol use disorders 2001–2012 (prior to outcome) (*N* = 11,078)	First-time hospitalization (or death, if no prior hospitalization) due to decompensated cirrhosis	HR: 1.92 (1.76–2.10) y
Alavi et al., 2018 [29]^b^	Scotland	Cohort study based on record linkage	1995–2014 (1995–2013 HCV notifications)	30,746 (1020)	Non-liver-related hospitalization due to alcohol use disorders 2001–2014 (prior to outcome) (*N* = 8757)	First-time hospitalization (or death, if no prior hospitalization) due to decompensated cirrhosis	HR: 3.88 (3.42–4.40) y
Harris et al., 2001 [19]	USA	Retrospective cohort study.	1968–1980	836 (142)	Loss of friends, family or job because of drinking; admitted to ever having a problem with alcoholism, medical records; sustained use of > 80 g/day (*N* = 149)	Liver cirrhosis	OR: 4.0 (2.1–7.7) y
Lim et al., 2014 [30]	USA	Case-control study	2002–2010	997 (27)	ICD-9 diagnosis for alcohol dependence/abuse recorded (*N* = 376)	Medical record–confirmed decompensated cirrhosis	OR: 2.46 (1.13–5.37) n
Marcellin et al., 2014 [31]	France	Case-control study	Not specified	304 (77)	Alcohol-related problems (physician’s report) (*N* = 41)	Advanced fibrosis	OR: 3.06 (1.42–6.60) y
Marcellin et al., 2015 [32]	France, Germany, Italy, Spain, UK	Case-control study	2006	1333 (438)	Chronic alcoholism (physician’s judgement) (*N* = 55)	Advanced fibrosis	OR: 2.51 (1.24–5.08) y
McDonald et al., 2010 [33]^b^	Scotland	Cohort study based on record linkage	1996–2006	15,878 (481)	Hospitalization due to alcohol use disorders or 100% alcohol-attributable disease 1996–2006 (prior to outcome) (*N* = 274)	First-time hospitalizations (or death, if no prior hospitalization) due to decompensated cirrhosis	HR: 5.50 (4.56–6.63) y
Nilsson et al., 2016 [34] ^e^	Sweden	Case-control analysis at baseline of a cohort study	2001–2010	284 (67 ascites, 15 variceal-bleeding, 9 encephalopathy)	Alcoholism or overconsumption of alcohol as stated in the medical records (*N* = 114)	Decompensated cirrhosis	OR: 3.24 (1.77–8.99)^e^ n
Nilsson et al., 2016 [34] ^c^	Sweden	Cohort study	2001–2010 (average follow-up 4.3 years)	284 (174)	Alcoholism or overconsumption of alcohol as stated in the medical records (*N* = 114)	Death (majority due to liver disease)	HR: 1.83 (1.34–2.51) y^d^
Schwarzinger et al., 2017 [8]	France	Retrospective cohort study based on record linkage	2008–2013	97,347 (15,630)	Hospitalization due to alcohol use disorders or 100% alcohol-attributable disease (*N* = 28,101)	First record of decompensated cirrhosis hospitalization	OR: 6.20 (5.85–6.58) y
Schwarzinger et al., 2017 [8]	France	Retrospective cohort study based on record linkage	2008–2013	97,347 (6677)	Hospitalization due to alcohol use disorders or 100% alcohol-attributable disease (*N* = 28,101)	Liver death (without liver transplantation)	OR: 7.63 (8.30–7.97) y
Sultanik et al., 2016 [35]	France	Retrospective cohort study	2006–2015	341 (136)	Either ICD-10 codes describing mental and behavioural states due to alcohol use disorders or 100% alcohol attributable	Hepatocellular carcinoma (35%) and/or end-stage liver disease	HR: 1.47 (1.02–2.13) y
Verbaan et al., 1998 [36]	Sweden	Case control	1991–1997	99 (20)	Use of > 80 g/day for at least 5 years; 92% of these were registered at Department of Alcohol Diseases, University Hospital, Malmö (*N* = 45)	Cirrhosis	OR: 11.8 (1.9–72.1) y
Wawrzynowicz-Syczewska et al., 2004 [37]	Poland	Cohort study	1988–2001	77 (22)	History of alcohol abuse (physician’s judgment) (*N* = 32)	Advanced fibrosis	OR: 10.00 (2.29–43.70) n

Highlighted areas were included in the main outcome variable: decompensated liver cirrhosis

*HR* Hazards Ratio, *OR* Odds Ratio

^a^ Risk relations are either Relative Risks, Hazard Ratios or Odds Ratios

^b^ The samples of the two studies [19, 33] overlap, with the methodology being slightly different (see definition of AUD). Only Alavi et al., 2018 [29] was included in the main quantitative meta-analysis

^c^ This outcome was not included into the second meta-analysis, as it was all-cause mortality, which is not a liver-specific outcome

^d^ The HR was estimated based on the methodology of Hamling et al. [26]

^e^ The combined OR was estimated by weighting the OR for ascites (OR: 4.39 (2.45–7.85)), variceal-bleeding (OR: 0.53(0.16–1.69)) and encephalopathy (OR: 5.50 (1.12–2.95)) by weighting the excess risks by the probability of risk occurrence

Lastly, we calculated the population-attributable fraction (PAF), i.e. the proportion of cases with liver disease progression that could have been avoided if no person with a chronic HCV infection had an AUD. The PAF was calculated using Formula 1 by combining data on the prevalence of AUD (P) with corresponding RRs [40]. All analyses were performed with R version 3.6.1 [41].

1 $$ \boldsymbol{PAF}=\frac{\boldsymbol{P}\left(\boldsymbol{RR}-\mathbf{1}\right)}{\mathbf{1}+\boldsymbol{P}\left(\boldsymbol{RR}-\mathbf{1}\right)} $$

## Results

Table 1 gives an overview of the studies and their characteristics. In total, studies including 286,641 people with chronic HCV infection fulfilled the inclusion criteria, of whom 63,931 (22.3%) qualified for an AUD.

**Fig. 2:**
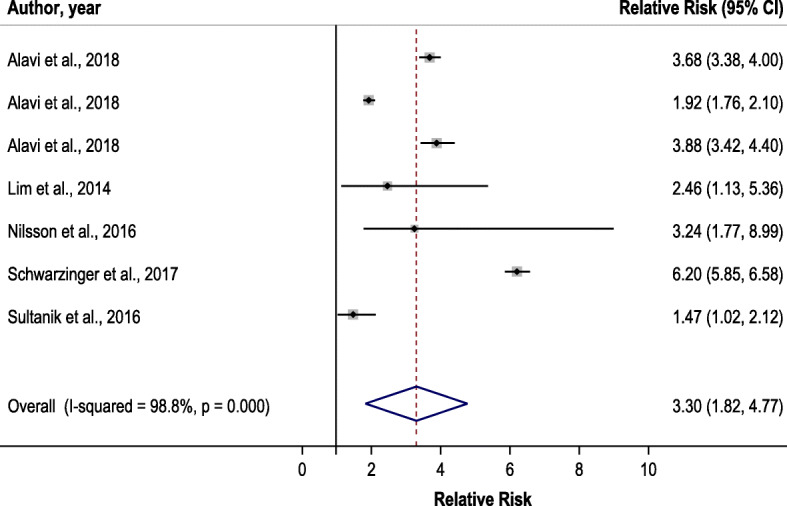
Forest plot for risk of decompensated liver cirrhosis associated with alcohol use disorder

In the main analysis, 268,114 people with chronic HCV were included, of whom 21,882 had decompensated liver cirrhosis (8.2%). A total of 63,335 people, or 23.6% of this sample, were identified with AUD, a proportion much higher than seen in the general population [2, 42]. Using decompensated liver cirrhosis as the outcome for the main analysis (based on *n* = 7 estimates), an AUD diagnosis was associated with a 3.3-fold risk for progression of liver disease among people with a chronic HCV infection (95% Confidence Interval (CI): 1.8–4.8), see Fig. 2). There was no significant difference between studies that were adjusted for important covariates and those that were not (*p*-value = 0.878). In terms of population attributable fractions, slightly less than 4 out ten cases of decompensated liver cirrhosis cases were attributable to AUD: 35.2% (95% CI: 16.2–47.1%).

As a secondary analysis, all estimates of liver disease progression were pooled, which yielded a similar overall effect of AUD as compared to the main analysis (*n* = 13 estimates; OR = 3.7; 95% CI: 2.2–5.3; see Supplementary Materials Figure S1). The risk difference between decompensated liver cirrhosis and the other indicators of liver disease progression was not significant (relative risk ratio: 0.6; 95% CI: 0.3–1.3). Again, adjustment for important covariates was not related to substantial reductions of the effect (*p*-value = 0.969). Again, this would be equivalent to about 40% of cases with liver progression being attributable to AUDs (attributable fraction: 39.3%; 95% CI: 21.9–50.4%).

In both analyses, substantial heterogeneity was identified using Cochran’s Q statistic ( [38]; main analysis for *n* = 7 estimates: Q (df = 6) = 507.3, *p* < .001; secondary analysis for *n* = 13 estimates: Q (df = 12) = 1184.5, p < .001), presumably associated with the large variation in sample sizes in the included studies (minimum = 77, maximum = 97,347). The I-square statistics also indicated substantial heterogeneity.

## Discussion

Before discussing the results and implications of our findings further, we would like to highlight the potential limitations.

### Limitations

One limitation to this review and meta-analysis is the reliance on aggregate data, which relies on the qualities of the underlying published studies, based on heterogeneous populations, different study designs and different statistical models, and in different historical periods of time [11]. Even though the populations were heterogeneous, almost all of the studies included are from high-income countries. Given the global load of alcohol-attributable liver cirrhosis burden [43, 44], we urgently need data from other regions of the world, especially from regions with a high prevalence of HCV infections such as Africa and Central Asia [2, 45], but also from countries in Eastern Europe where prevalence of HCV infections and of AUDs are high (e.g., Moldova, Georgia [46]).

Also, the largest studies [8, 29] relied on medical hospital records of AUD, which likely underestimated the true prevalence, as this disorder is highly stigmatized [47] and neither necessarily disclosed nor recorded in hospitals or healthcare settings (for a wider discussion, see [48, 49]), even for 100% alcohol-attributable disorders [50, 51]. However, the bias introduced by underestimating the prevalence of AUDs is conservative; the attributable fractions would likely be higher with higher prevalence (for formulas, see [40]). Additionally, relying on hospital records for the largest studies removes potential biases due to self-report of AUDs [52].

Another potential limitation involves the exclusion of studies where AUDs could only have been inferred by a mention of chronic heavy drinking or other drinking behaviours closely related to AUDs. On the one hand, heavy drinking is a key characteristic of AUDs [20]. To give one example, there is a high likelihood that lifetime drinkers with more than 175 g pure alcohol consumed daily—such as in the study of Corrao and colleagues [53]—would qualify for AUDs had this condition been measured with validated instruments. One the other hand, it is hard to draw a threshold. In the same study by Corrao and colleagues, the following thresholds were used to indicate the drinking level: 50 g, 75 g, 100 g, 125 g and 150 g pure alcohol per day. It is not clear which of these drinking-level categories would indicate AUD. Thus, while AUDs constitute a common medical diagnosis, the use of this diagnosis—which is clinically relevant and can be used in health services research—in epidemiological research may lead to biases, as the active ingredient in disease progression—ethanol—is only indirectly assessed (see also [7, 54]). Another aspect of patterns of drinking deserve mentioning. These patterns – especially the prevalence of heavy episodic drinking – differ vastly between the countries examined here [55]. It has been shown that a pattern of daily heavy drinking is most detrimental for worsening of liver disease [56, 57], for daily drinking is less prevalent among heavy drinkers in countries like Poland or Scotland, compared countries like France, to mention just three of the countries in our sample. Without measuring patterns of drinking at the individual level, variation is introduced into our results. Future research should not only rely on wide categories such as AUD [6], but should measure drinking level and patterns.

As we wanted to conduct a meta-analysis with a narrow outcome – decompensated liver cirrhosis, we defined our search terms excluding wider definitions such as hepatocellular carcinoma. Indeed, we achieved this goal, and only in one study [35], a minority of cases included hepatocellular carcinoma. This does not mean, however, that AUDs do not causally impact on hepatocellular carcinoma in people with chronic HCV infections. As Schwarzinger and colleagues demonstrated in the national French hospital cohort comprising 6404 patients with hepatocellular carcinoma [8], AUDs were associated with a fourfold-increased risk (4.23; 95% CI: 3.99–4.49).

### Alcohol use disorders as a key determinant of liver disease progression

Our results show that AUDs are quite common among people with chronic HCV infections, and that they are a key determinant for worsening of liver disease. Our design did not allow us to answer the question of whether alcohol use or AUDs were the only factor in disease progression (see [15]); however, other research seems to indicate that HCV in people with AUDs also showed increased disease progression (e.g., [6, 58, 59]) and, thus, there seems to be an interaction effect of alcohol use and HCV infection. There are also plausible biological pathways, such as increased viral replication and altered immune response [60].

Our design also does not answer the question regarding a dose-response relationship for alcohol use, i.e., whether all levels of alcohol use are detrimental for liver disease progression (see [16, 61]). However, we can clearly state that AUDs, with their high levels of alcohol consumption, produce a markedly worsened progression for liver diseases, and were responsible for about 40% of all these complications in the large cohorts underlying our study (see [62, 63], for further discussion). Such a high attributable fraction also calls into question the compartmentalization of liver cirrhosis into subtypes/categories [4, 5].

## Conclusions

AUDs are relatively frequent in people with chronic HCV infections and contributed markedly to progression of liver disease. Two main conclusions result: despite the current clinical compartmentalization, alcohol use and AUDs should be assessed in patients with liver cirrhosis of any etiology (see also [64] for the dose-response relationship for alcohol use on any kind of liver cirrhosis), and irrespective, if the HCV infection has been treated successfully of not. This is even true for so-called “non-alcoholic” liver disease categories, where AUDs do not play a role by definition, but alcohol use still may [65]. But assessment is not sufficient, AUDs need to be treated by either achieving full abstinence (the best outcome of alcohol interventions for any kind of liver disease [66]) or, if this is not possible, reducing consumption to the highest degree possible [67–69].

## Supplementary information

**Additional file 1.** Supplementary materials.

**Additional file 2: Figure S1.** Forest plot for risk of negative course of liver disease associated with alcohol use disorder.

## Data Availability

All data has been derived from published studies.

## Abbreviations

AUD/AUDs: Alcohol Use Disorder(s)
CI: Confidence Interval
HCV: Hepatitis C Virus
PAF: Population-attributable fraction
PRISMA: Preferred Reporting Items for Systematic Reviews and Meta-Analyses
RE model: Random-effects model
WHO: World Health Organization
