# Supplementary material for: Alcohol use disorders and the risk of progression of liver disease in people with hepatitis C virus infection – a systematic review
Source: Subst Abuse Treat Prev Policy. 2020 Jun 30;15:45. doi: 10.1186/s13011-020-00287-1 (PMC7325038; doi:10.1186/s13011-020-00287-1)
Supplement: Supplementary file 1 — Additional file 1. Supplementary materials. [file 13011_2020_287_MOESM1_ESM.docx]

# Supplementary materials:

# Alcohol use disorders and the risk of progression of liver disease in people with Hepatitis C virus infection – a systematic review

Laura Llamosas-Falcón, Kevin D. Shield, Maya Gelovany, Jakob Manthey, Jürgen Rehm

## Search strategy for epidemiological studies on the relationship between alcohol use and progression of liver disease due to HCV, using Ovid Medline databases (22.12. 2019)

| **#** | **Search Statement** | **Results** |
| --- | --- | --- |
| 1 | exp Hepatitis C/ | 62540 |
| 2 | incidence/ or epidemiologic research design/ or epidemiologic study characteristics/ or epidemiological monitoring/ | 261021 |
| 3 | exp Liver Diseases, Alcoholic/ or exp Alcoholism/ or exp Liver Cirrhosis/ | 164121 |
| 4 | 1 and 2 and 3 | 358 |
| 5 | epidemiologic studies/ or case-control studies/ or cohort studies/ or follow-up studies/ or longitudinal studies/ or prospective studies/ or retrospective studies/ | 2152739 |
| 6 | 1 and 2 and 5 | 989 |
| 7 | incidence/ or prevalence/ or epidemiologic research design/ or epidemiologic study characteristics/ or epidemiological monitoring/ | 516449 |
| 8 | exp Liver Diseases, Alcoholic/ | 14381 |
| 9 | longitudinal studies/ or prospective studies/ or retrospective studies/ | 1392057 |
| 10 | 2 or 5 or 7 or 9 | 2489399 |
| 11 | exp Alcohol Drinking/ | 67182 |
| 12 | 8 or 11 | 80434 |
| 13 | 1 and 10 and 12 | 558 |
| 14 | 1 or 10 or 11 or 12 | 2592515 |
| 15 | from 13 keep 3,11-12,16-21,27 | 10 |
| 16 | 2 or 5 | 2298781 |
| 17 | 1 and 3 and 16 | 3041 |
| 18 | 3 or 11 | 218616 |
| 19 | 2 or 5 or 9 | 2298781 |
| 20 | 1 and 18 and 19 | 3171 |
| 21 | 2 or 5 | 2298781 |
| 22 | 1 and 3 and 21 | 3041 |
| 23 | 8 or 11 | 80434 |
| 24 | 1 and 21 and 23 | 467 |

## Figure S1: Forest plot for risk of negative course of liver disease associated with alcohol use disorder

Legend: Relative Risk point estimates and 95% confidence intervals for studies included in the sensitivity analyses. References for all studies see main text.
