# Supplementary material for: Alcohol use disorders and the risk of progression of liver disease in people with hepatitis C virus infection – a systematic review
Source: Subst Abuse Treat Prev Policy. 2020 Jun 30;15:45. doi: 10.1186/s13011-020-00287-1 (PMC7325038; doi:10.1186/s13011-020-00287-1)
Supplement: Supplementary file 2 — Additional file 2: Figure S1. Forest plot for risk of negative course of liver disease associated with alcohol use disorder. [file 13011_2020_287_MOESM2_ESM.pdf]

Figure S1: Forest plot for risk of negative course of liver disease associated with alcohol use disorder

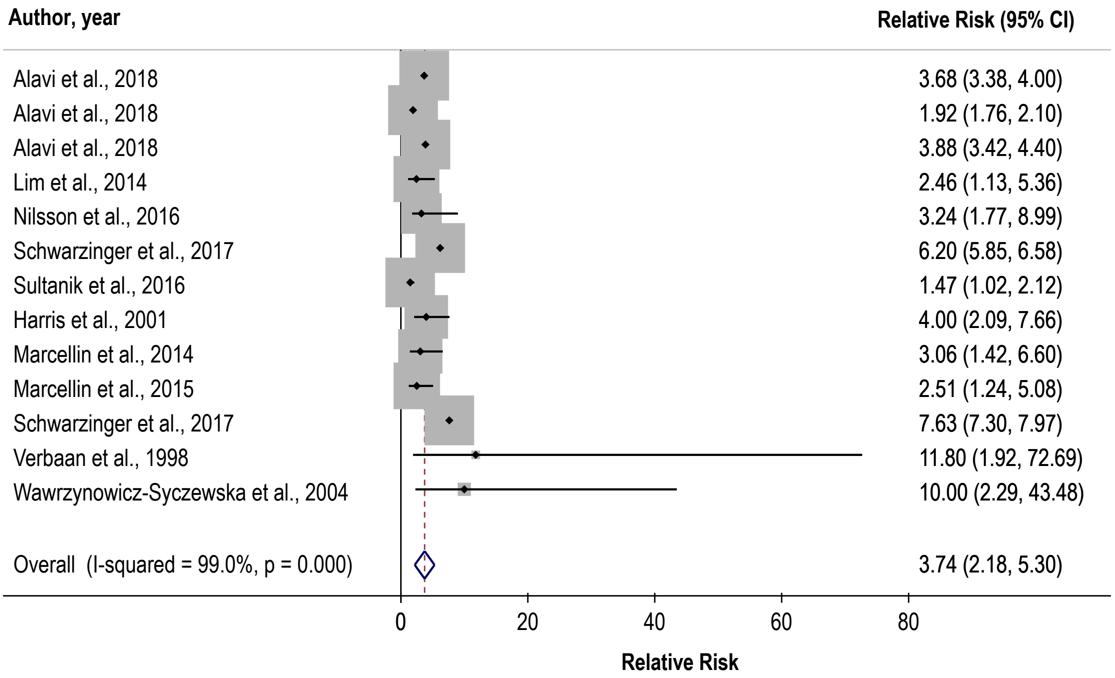

Legend: Relative Risk point estimates and 95% confidence intervals for studies included in the sensitivity analyses. References for all studies see main text.
